# First report of *Meloidogyne graminis* on golf courses turfgrass in Brazil

**DOI:** 10.1371/journal.pone.0192397

**Published:** 2018-02-07

**Authors:** Samara Azevedo de Oliveira, Cláudio Marcelo Gonçalves de Oliveira, Carla Maria Nobre Maleita, Maria de Fátima A. Silva, Isabel Maria de Oliveira Abrantes, Silvia Renata S. Wilcken

**Affiliations:** 1 Universidade Estadual Paulista, Faculdade de Ciências Agronômicas, Campus de Botucatu, Rua José Barbosa de Barros, Botucatu, São Paulo, Brazil; 2 Laboratório de Nematologia, Instituto Biológico, Campinas, São Paulo, Brazil; 3 CIEPQPF—Chemical Process Engineering and Forest Products Research Centre, Chemical Engineering Department, University of Coimbra (UC), Rua Sílvio Lima, Pólo II, Pinhal de Marrocos, Coimbra, Portugal; 4 CFE—Centre for Functional Ecology, Department of Life Sciences, UC, Calçada Martim de Freitas, Coimbra, Portugal; Stony Brook University, UNITED STATES

## Abstract

Plant-parasitic nematodes of the genus *Meloidogyne*, known as root-knot nematodes (RKN), have an important economic impact on golf course turfgrasses. The most prevalent RKN species associated with grasses are *M*. *chitwoodi*, *M*. *graminicola*, *M*. *graminis*, *M*. *incognita*, *M*. *marylandi*, *M*. *microtyla*, *M*. *minor*, *M*. *naasi* and *M*. *sasseri*. In 2010, slight thickening of the roots and RKN females with unusual features were observed in turfgrass roots on golf courses in Araras, São Paulo state, Brazil. This population (MgARA) was maintained in the lab and studied including morphological, morphometrical, biochemical and molecular markers. Morphology and morphometry were variable and not useful for identification, although perineal pattern morphology showed highly similarity with *M*. *graminis* description. Concerning to biochemical characterisation, the esterase phenotype Mg1, characterised by a very slow and fainter band, was detected in some protein homogenates. Regarding to molecular analysis, D2-D3 region of 28S rDNA gene and cytochrome oxidase subunit II region from mitochondrial DNA were amplified by PCR and sequenced. Phylogenetic analysis revealed that the Brazilian isolate, found associated with turfgrass, grouped with *M*. *graminis* isolates (98–99% bootstrap; variation of 8–11 and 0–24 bp, respectively), close to *M*. *marylandi*, supporting its identification as *M*. *graminis*. This is the first report of *M*. *graminis* on golf courses in Brazil.

## Introduction

The turfgrass industry is expanding in Brazil and the biggest consumer market for turfgrass is the sports industry, mainly soccer fields and golf courses. The quality of the turf in these areas is crucial, especially on golf courses, where any imperfection can affect the outcome of the sport [1]. There are about 110 official state-owned golf courses in Brazil, in addition to many private courses, each measuring ca. 50 ha [2].

Plant-parasitic nematodes (PPN) are one of the most damaging pathogens to golf courses, affecting health, quality and maintenance of turfgrass. Nematodes that are considered the most damaging PPN on turfgrasses in the United States, include *Anguina pacificae*, *Criconemella* spp., *Heterodera* spp., *Helicotylenchus* spp., *Hoplolaimu*s spp., *Meloidogyne* spp., *Pratylenchus* spp., *Trichodorus* spp., *Mesocriconema ornatum* and *Belonolaimus longicaudatus* [3,4]. Root-knot nematodes (RKN), *Meloidogyne* spp., are distributed worldwide and parasitise a wide range of economically important plants, causing serious damage to golf course turf. RKN species associated with turfgrasses are *M*. *chitwoodi*, *M*. *graminicola*, *M*. *graminis*, *M*. *incognita*, *M*. *marylandi*, *M*. *microtyla*, *M*. *minor*, *M*. *naasi* and *M*. *sasseri* [4,5]. In Brazil, an unidentified *Meloidogyne* population was found in turfgrass roots in Parana State; *M*. *graminicola* associated with rice roots in Santa Catarina and Rio Grande do Sul States; and *M*. *incognita* and *M*. *javanica* in several forage grasses around the country [6,7].

*Meloidogyne graminis* was identified as a parasite of turfgrass for the first time in 1959 in Florida, by Sledge [8]. This species is associated with several plant species, however, and according to available information, has a host range restricted to grasses. The damage caused by this RKN is characterised by the presence of large circular areas of dead or dying grass and chlorosis causing a high economic impact [9]. This species has a large distribution in the United States (Alabama, Arizona, Arkansas, California, Florida, Georgia, Hawaii, Kansas, Maryland, Nevada, New England, North Carolina, South Carolina, Tennessee, Texas and Virginia states), but with a limited distribution worldwide [5,8,9,10]. Meanwhile, this species was already reported in India, Libya, China [9,11,12], in Europe (Germany and The Netherlands) associated with grasses and cereals [9,13], and in South America (Venezuela), causing also damage to turfgrasses of golf courses [14]. So far there are no reports of *M*. *graminis* in Brazil.

The impact of *M*. *graminis* on golf courses and the need of effective nematode management programs and monitor its distribution and spread, reinforce the importance of a reliable and rapid diagnosis of this nematode species. For the accurate diagnosis of *Meloidogyne* species, in addition to morphology, morphometry and biochemistry, the use of molecular markers is also required [15]. The regions of the genome commonly used for the characterisation of RKN species are the ribosomal DNA (rDNA- 18S, 5.8S and 28S genes, and spacer regions, ITS-1 and ITS-2) and the mitochondrial DNA (mtDNA) particularly the region between genes encoding cytochrome oxidase subunit II (COII) and 16S rRNA [10,16]. McClure et al. [10], based on sequence and phylogenetic analyses of 18S rDNA, D2-D3 region of 28S rDNA, internal transcribed spacer-rRNA, and mtDNA gene sequences, have identified RKN in golf courses from the western United States. The mtDNA-PCR-RFLP analysis showed to be an efficient methodology to discriminate *M*. *graminis* from *M*. *marylandi* with the restriction enzyme *Ssp*I [10]. More recently, four RKN (*M*. *graminis*, *M*. *incognita M*. *marylandi* and *M*. *naasi*) from turfgrasses in North Carolina were identified through a combined analysis of DNA sequencing (rDNA 18S, ITS and 28S D2-D3) and PCR by species-specific primers [5].

This research reports for the first time *M*. *graminis* associated to damage of golf courses in Araras, São Paulo, Brazil. Morphometrical, morphological, biochemical and molecular markers were used to characterise and confirm the identification of this isolate. The relationship with other RKN was also performed based on analyses of sequences from the D2-D3 region of 28S rDNA gene and mtDNA COII.

## Material and methods

### Nematode

Yellow patches, reduced or stunted growth and thinning of the turfgrass foliage, and reduced and damaged root systems were observed in a golf course of Araras, in the state of São Paulo, Brazil. However, typical galls were not detected in roots and only a slight thickening of the roots in the nematode feeding site region was observed. Chlorotic turfgrasses plants were sent to the Nematology Laboratory of Agricultural Sciences University/UNESP Botucatu (SP) and the presence of *Meloidogyne* females with unusual features were found in roots. Second-stage juveniles (J2) and eggs were extracted from several infected roots using the Coolen & D'Herde method [17] and inoculated in turfgrass pots. This *Meloidogyne* isolate (MgARA) was maintained in a green house. For optimal egg production and long-term maintenance, the nematodes were transferred every two months to new hosts.

Morphological, morphometrical, biochemical and molecular characterisation of this isolate was made from the turfgrass culture.

### Morphometric and morphological characterisation

Males (30) and J2 (40) were extracted from infected turfgrass roots and eighteen characters were observed and measured (Table 1). Perineal patterns were also prepared for light microscope studies according to Taylor & Netscher [18].

**Table 1:** Morphometrics of males and second-stage juveniles of *Meloidogyne* sp. isolate MgARA.

Characteristic	Males (*n* = 30)	Second-stage juveniles (*n* = 40)
*Linear (μm)*		
Body length	1269.7 ± 240*	413.7 ± 22.6*
	(900–1705)	(365–475)
Greatest body width	36.6 ± 5	16.0 ± 1.2
	(25–47.5)	(13–18)
Body width at stylet knobs	16.3 ± 2	9.3 ± 0.5
	(13.7–20)	(8–10)
Body width at excretory pore	27.9 ± 5	14.3 ± 1
	(22.5–42.5)	(12–16)
Body width at anus	23.3 ± 3	10.8 ± 0.9
	(17.5–30)	(9–13)
Stylet length	15.5 ± 1	10.3 ± 0.9
	(13–17)	(9–12)
Dorsal esophageal gland orifice	3.4 ± 1	3.2 ± 0.5
	(2.5–5)	(2–4)
Excretory pore to anterior end	132.9 ± 15	71.9 ± 5.9
	(100–152.5)	(62–90)
Anterior end to metacorpus	74.6 ± 9	49.2 ± 3.1
	(61–90)	(40–55)
Metacorpus length	17 ± 3	12.5± 1.4
	(13–22)	(10–15)
Metacorpus width	10.9 ± 1	8.4 ± 1.5
	(10–14)	(7–8)
Tail length	26.3 ± 5	68.5 ± 4.5
	(20–37.5)	(52–76)
Anus to gonad primordium	―	93 ± 5.1
		(85–98)
Tail terminus length	―	13.2 ± 1.8
		(10–17)
Spicule length	30.7 ± 2	―
	(27–34)	
Gubernaculum length	6.3 ± 1	―
	(4–8)	
*Ratio*		
a = Body length/body width	34.8 ± 6	25.8 ± 2.5
	(23.1–45.4)	(21.1–31.6)
c = Body length/tail length	0.02 ± 0	6 ± 0.4
	(0.01–0.04)	(5.5–7.7)
c' = tail/body width at anus	1.1 ± 0.1	6.3 ± 0.7
	(0.8–1.4)	(4.7–8.1)
Excretory pore/BL×100	10.8 ± 2	17.4±1.7
	(7.3–15.8)	(13.7–22.5)

*Values are mean ± standard deviation (range).

Additionally, males, females and freshly hatched J2 were prepared for scanning electron microscopy (SEM), as described by Abrantes and Santos [19]. Specimens were mounted on stubs, coated with gold (200Å), viewed, and photographed with an EDAX PEGASUS X4M (Materials Centre of the University of Porto, Portugal).

### Biochemical characterisation

Mature females (10/tube) were handpicked from infected turfgrass roots and transferred to micro-hematocrit tubes containing 5 μL of extraction buffer (20% [wt/vol] sucrose, 2% [vol/vol] Triton X-100 and 78% [vol/vol] distilled water). The specimens were macerated with a pestle and the tubes stored in a freezer (-20°C) until electrophoresis. Esterase electrophoresis was performed according to Oliveira et al. [20].

### Molecular characterisation

#### DNA extraction and PCR

Genomic DNA was extracted from J2, through a modified Holterman et al. protocol [21]. Nematodes were transferred, individually, to a glass slide with 5 μL of Holterman Lysis Buffer (HLB), containing proteinase K 800 μg/mL, β-mercaptoethanol 1% (v/v), 0.2 M NaCl, and 0.2 M Tris-HCl, at pH 8, and cut into three parts with a sharp scalpel blade. The nematodes and the lysis buffer were transferred to a sterile 0.2 mL PCR tube containing 45 μL HLB. Samples were incubated at 65°C for 2 h followed by 99°C for 5 min, and then stored at -20°C prior to PCR.

The D2-D3 region of the 28S rDNA gene and mtDNA COII were selected to carry out the molecular characterisation of MgARA isolate as described in Al-Banna et al. [22]. Total reaction volume of 25 μL contained 12.5 μL of Gotaq Hot Start, 3.5 μL of nuclease free water, 1 μL of each primer (10 mM) and 1 μL of DNA. PCR were performed using the following conditions: 95°C for 3 min; followed by 35 cycles of 95°C for 1 min, 68°C for 1 min, 72°C for 1 min; and a final extension at 72°C for 10 min. Primers D2A (5′-ACAAGTACCGTGAGGGAAAGTTG-3′) and D3B (5′-TCGGAAGGAACCAGCTACTA-3′) [23] were used for the D2-D3 region. For the mtDNA COII gene was used the C2F3 (5′-GGTCAATGTTCAGAAATTTGTGG-3′) and MRH106 (5′-AATTTCTAAAGACTTTTCTTAGT-3′) primers [24,25]. The PCR products were analysed on a 1.0% agarose gel in 1×TAE buffer stained with ethidium bromide, and viewed under UV light.

#### Sequencing and phylogenetic analyses

PCR products were purified using the Ultraclean PCR Clean-Up Kit (MO Bio Laboratories, Carlsbad, CA, USA) according to the manufacturer's instructions. DNA sequencing of amplified regions was conducted in the Biochemistry Phytopathological Laboratory of the Biological Institute, São Paulo, Brazil, according to the procedures described by Oliveira et al. [26]. The sequences of each region were deposited in the GenBank as KY131985 (D2-D3 region of the 28S rDNA) and KY131986 (mtDNA COII).

Sequences were compared with sequences of other PPN species, obtained by searching the GenBank nucleotide database (http://www.ncbi.nlm.nih.gov) using the software BioEdit; and similarity was evaluated using BLASTN 2.2.19+ program [27].

Phylogenetic analyses were conducted in MEGA 6 [28]. Hasegawa-Kishino-Yano model [29] showed the lowest BIC scores (Bayesian Information Criterion) and was considered to describe the substitution pattern the best. One thousand bootstrap replicates were performed to test the support of each node on the trees [28]. *Pratylenchus vulnus* was selected as outgroup for the D2-D3 region (JQ003992.1), and *M*. *enterolobii* for the mtDNA COII region (FJ159617.1). Outgroup taxa for each dataset were selected according to previously published data [10].

## Results

### Morphometric and morphological characterisation

Morphometrics of *Meloidogyne* sp. isolate MgARA males and J2, found in golf courses in Araras, are presented in Table 1.

**Fig 1:**
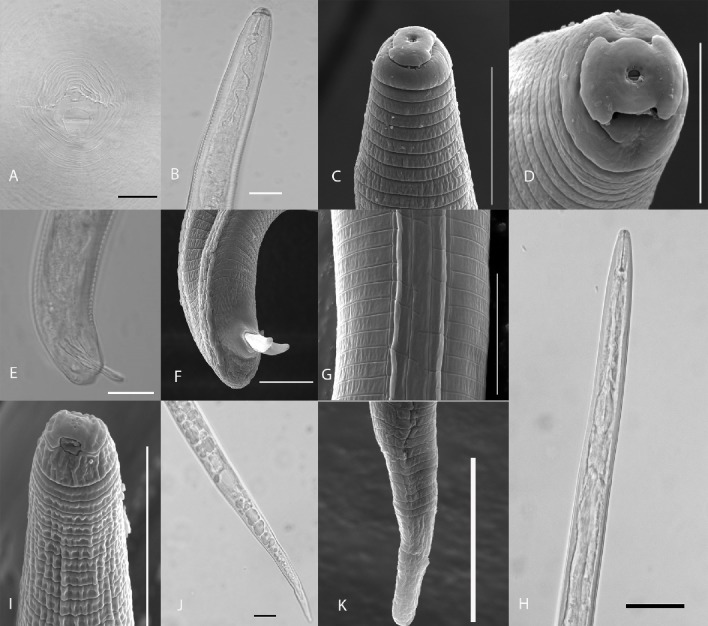
Meloidogyne graminis. Light (A, B, E, H, J) and scanning electron microscope (C, D, F, G, I, K) photographs. Females: A, perineal pattern. Males: B, C, anterior region in lateral view; D, head region showing inner labial sensilla; E, F, posterior region in lateral view; and G, lateral lines. Second-stage juveniles: H, anterior region; I, head region; and J, K tail region. Scale bars: A, B, E, H, J—20 μm; C, F, K—10 μm; D, G, I—5 μm.

Females with a whitish oval body and an annulated and thin thick cuticle; had a protruding elongated neck; lip region variable in exact shape but in general with circumoral elevation; stylet knobs rounded posteriorly; a well-developed pharynx with an elongate, cylindrical procorpus; and a large and rounded metacorpus with well-developed valves. In the posterior end was observed the presence of two ovaries, vulva and anus situated posteriorly on a protrusion of the body. Perineal pattern rounded to oval shaped, well defined, high and rounded to squared dorsal arch with coarse striae. Lateral field incisures distinct and marked by the junction of dorsal and ventral striae; tail terminus area free of striae and absence of punctuations near anus (Fig 1A).

Males had an elongated and vermiform body tapering gradually at the ends, striated cuticle and a lateral field with four incisures. In the anterior region a robust stout stylet is present with prominent rounded knobs; a well-developed cephalic framework and a median bulb elongated with well-developed valves. At the posterior region was observed the spicules, a slender gubernaculum, curved ventrally, and, in general rounded tail end; one testis (Fig 1B–1G).

J2 exhibited a vermiform and cylindrical body tapering to the posterior end; cuticular annulation well marked, lateral field with four lines; a straight tail gradually tapering until the end of body; a hyaline tail terminus distinct, relatively long and an evident anus. In the anterior region, the cephalic framework is indistinct, it was observed a small and delicate stylet with rounded knobs; DGO region distinct as the excretory pore and a median bulb elongated with well-developed valves (Fig 1H–1K).

### Biochemical characterisation

**Fig 2:**
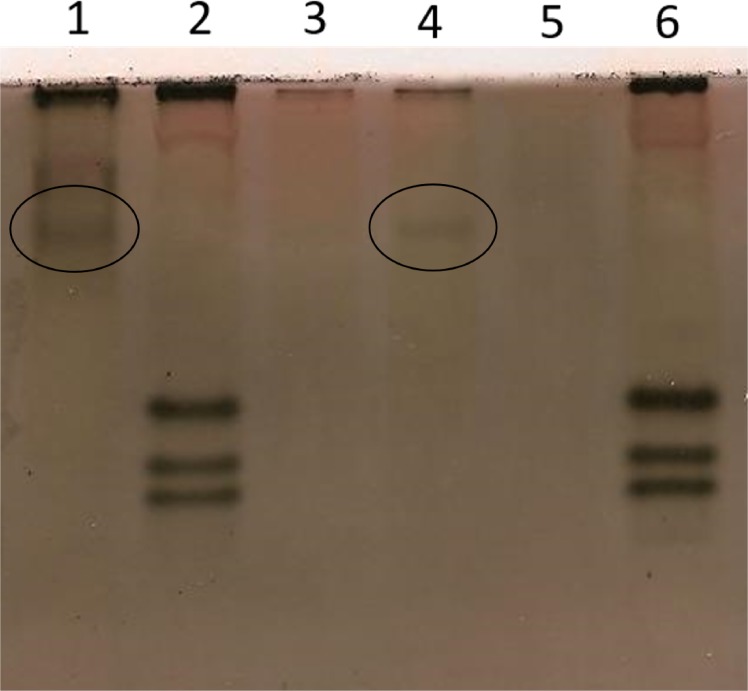
Polyacrylamide gel stained for esterase activity. Mg1, *Meloidogyne* sp. isolate MgARA; J3, *M*. *javanica* (reference isolate). Protein homogenate from ten egg-laying females was applied to each well.

Concerning to biochemical analysis, esterase electrophoresis was performed to complement MgARA isolate characterisation. The esterase phenotype Mg1 was observed in two samples (Fig 2), which exhibited a single band with a very slow migration (Rm: 0.47). In turn, in the *M*. *javanica* isolate, included as reference isolate, three bands of esterase activity were detected (J3, Fig 2). The analysis was performed three times and the results were reproducible.

### Molecular characterisation

**Fig 3:**
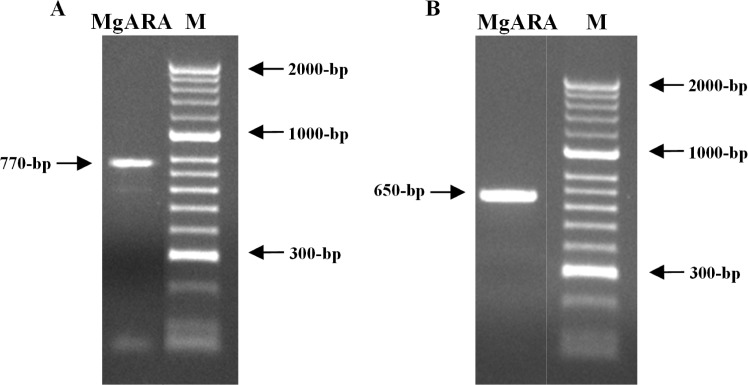
DNA amplification products obtained from *Meloidogyne* sp. isolate MgARA to (A) D2-D3 region of the 28S rDNA gene and (B) mtDNA COII. M—DNA marker (HyperLadder II; Bioline).

The D2-D3 region of the 28S rDNA gene and mtDNA COII, amplified with the primer sets D2A/D3B and C2F3/MRH106 from purified DNA extracted from MgARA J2 yielded single fragments of ca. 770 and 650 bp, respectively (Fig 3).

Based on D2-D3 expansion segments of the 28S rRNA gene sequences, the studied isolate showed high similarity with isolates of *M*. *graminis* and *M*. *marylandi* from the United States. MgARA sequence had 99% affinity with published *M*. *graminis* (KP901075) sequence and 98% with *M*. *marylandi* (JN019350).

However, based on mtDNA COII region, the sequence obtained for the MgARA isolate were identical to a *M*. *graminis* sequence available in the GenBank (JN241915), considering E-value of 0.0 and similarity of 100%. When compared with *M*. *marylandi* (KC473862), the homology was only 84% of similarity. At the species level, the majority of the sequences of *M*. *graminis* (including MgARA) had variation of 0 to 24 bp, but the pairwise sequence comparison between MgARA isolate and *M*. *marylandi* differed by 70 nucleotides deletion or insertion.

**Fig 4:**
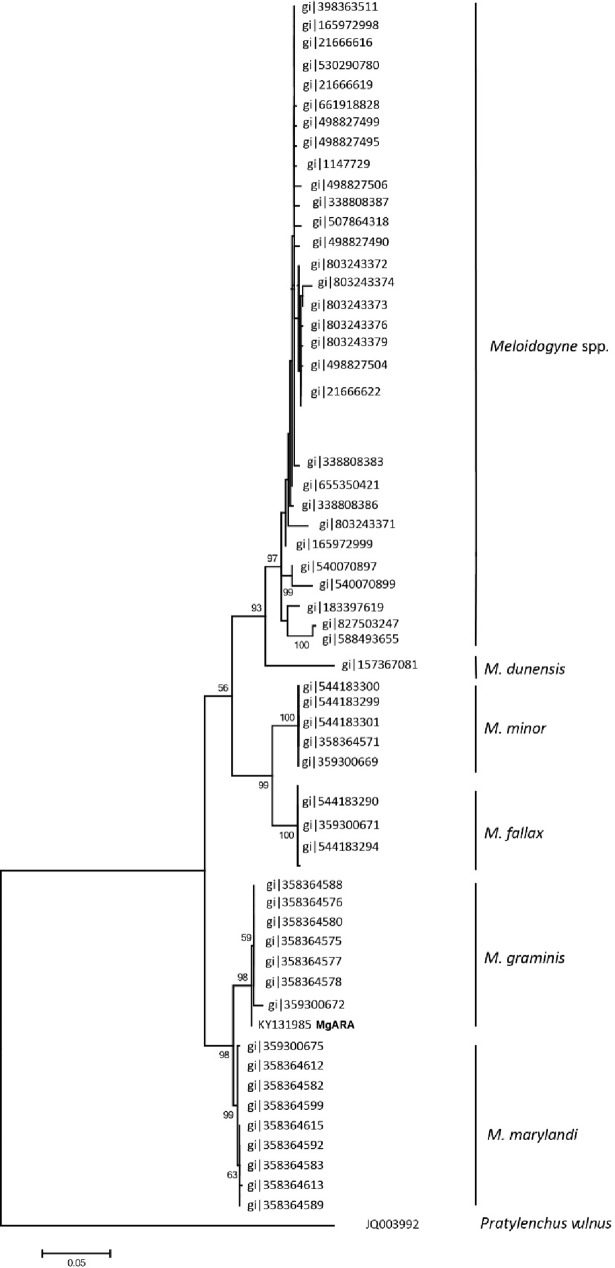
Phylogenetic relationship based on the alignment of sequences of D2-D3 region of 28S rDNA gene of *Meloidogyne* sp. isolate MgARA with available sequences of other *Meloidogyne* species. *Pratylenchus vulnus* was used as outgroup.

In total, 62 sequences were included in the phylogenetic analysis of D2-D3 expansion segments of the 28S rRNA gene. A highly-supported clade with *M*. *graminis* and the isolate MgARA was distinguished in the consensus tree (Fig 4), and was a sister taxon to *M*. *marylandi*, confirming that the Brazilian isolate MgARA, found associated with turfgrass in São Paulo State, belongs to *M*. *graminis* species. Intraspecific sequence variation for *M*. *graminis* species reached 1.0% (8–11 bp). Interspecific sequence variation between *M*. *marylandi* and *M*. *graminis* was 2.0% (14 bp) (Fig 4).

**Fig 5:**
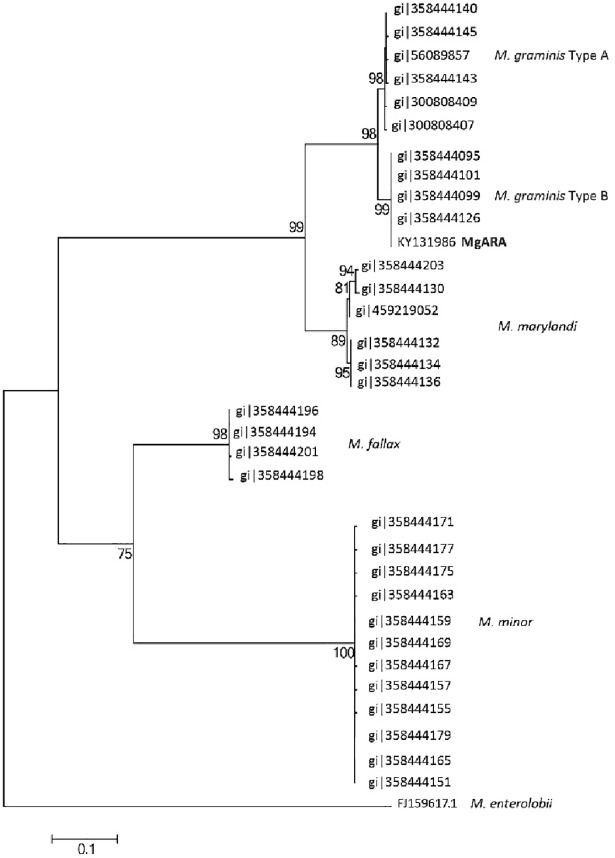
Phylogenetic relationship based on the alignment of sequences of mtDNA COII region of *Meloidogyne* sp. isolate MgARA with available sequences of other *Meloidogyne* species. *M*. *enterolobii* was used as outgroup.

The phylogenetic tree obtained from analysis of mtDNA COII region involved 35 sequences and showed a highly-supported clade based on *M*. *graminis* and *M*. *marylandi* sequences. *Meloidogyne graminis* formed two robust subclades: type A and B (98 and 99% bootstrap, respectively). The Brazilian isolate MgARA remained in the same group as *M*. *graminis* type B (Fig 5).

## Discussion

Plant-parasitic nematodes, particularly *Meloidogyne* spp., are among the most damaging and difficult to manage agronomic pests. The identification of the RKN species is fundamental to effective crop management and quarantine control. However, its identification is difficult due to the high number of described species, morphological similarity between species and intraspecific variability.

Different tools have been used to have an accurate identification of *Meloidogyne* species, among them, biochemical and molecular approaches are increasing as a routine practice in nematology laboratories. These procedures alone may not be sufficient for the diagnosis of uncommon RKN species, therefore, should be considered together [15,27,30].

Due to the combination of the biochemical and molecular approaches, the RKN MgARA isolate, associated with turfgrass in a golf course in Araras, São Paulo State, was identified as *M*. *graminis*. This is the first time that this species is reported in Brazil.

Typical RKN symptoms on the turfgrass field infested with *M*. *graminis* were observed, such as patches of small and chlorotic plants and reduced turfgrass quality, affecting the roll of the golf ball. Furthermore, the turf had a bad response to regular maintenance procedures and became unable to withstand normal levels of wear and tear during play [31]. The typical RKN galls were not observed, only a slight thickening of the roots was detected which agree with Mitkowski [32].

Morphometrically, *M*. *graminis* MgARA isolate (males and J2) presented a high variability and differed from the original description and from other described isolates [8,10,14]. It was clear that J2 of *M*. *graminis* and *M*. *marylandi* overlap morphometrically. Thus, the use of morphometrical characteristics could lead to mistaken identification.

Instead of morphometric characters, morphological features were considered, in the past, one of the most useful characters in the identification of *Meloidogyne* species [33]. The morphology of males and J2 of the *M*. *graminis* isolate was similar to the species description [8]. Despite the similarity with *M*. *microtyla*, morphology of perineal patterns of the MgARA isolate was similar to the described patterns of *M*. *graminis*: coarse striae, well defined squared dorsal arch, lateral lines distinct, and forking of dorsal and ventral striae [34]. *Meloidogyne microtyla* striae is not as coarse of that of *M*. *graminis* ([34]. Nevertheless, the morphology of the perineal patterns should not be the only characteristic considered in RKN diagnosis, due to the occurrence of a high inter and intra-specific morphological variability and it should be used as a complement to biochemical and molecular studies [35]. Integrative and polyphasic taxonomy is the securest path to nematodes diagnosis [36].

The esterase phenotype Mg1, characterised by a very slow and fainter band, was detected in some protein homogenates (10 egg-laying females/tube) of the MgARA isolate. This phenotype has been previously reported by Brito et al. [30] from *M*. *graminis* isolated from the ornamental plants *Stenotaphrum secundatum*. Because the specific and unique band of MgARA can vary with the amount of protein and time of staining, like in *M*. *exigua* [37], to increase the signal intensity a higher number of females is needed.

DNA barcoding allowed a clearly identification of the MgARA isolate as *M*. *graminis*. Sequences from the D2-D3 region of 28S rDNA gene and mtDNA COII gene were selected to characterise the MgARA isolate, demonstrating that these DNA regions have enough information to provide an accurate diagnosis: MgARA sequences showed 99% similarity rate for D2-D3 and 100% for mtDNA COII with USA populations. The first application of DNA barcode technology in Brazil was applied on *Pratylenchus penetrans* identification [38]. A similar methodology was used in the present study, demonstrating that this technology can be used with success to identify *M*. *graminis* isolates.

Taking into account the results of the phylogenetic study of mtDNA COII region, McClure et al. [10] identified two haplotypes for *M*. *graminis*: haplotypes A and B. Brazilian *M*. *graminis* isolate (MgARA) was grouped with isolates from haplotype B, which suggest that this species was potentially originated from California, Arizona or Texas. Moreover, a close relationship between *M*. *graminis* and *M*. *marylandi* was also reported; however, these species can be successful differentiated by molecular studies.

In Brazil, there is no studies about the abundance and diversity of PPN associated with turfgrasses. The difficulty to recognize the symptoms exhibited by turfgrasses infected with RKN, which can be easily confused with damage associated with poor nutrition or injury caused by bacteria, pathogenic fungi and/or virus, and the fact that the majority of the turfgrasses applied in golf courses and soccer fields are imported from United States, are the most important reasons that explain the absence of these studies. However, this work showed that nematodes of the genus *Meloidogyne* can be found and potentially cause damage in golf courses whereby sanitizer authorities must be alert to the importation of turgrasses. National and international measures should be established to decrease the risk of spread and introduction of *M*. *graminis* species into regions where it does not exist. The use and transport of clean, healthy, nematode-free planting material is a prerequisite for limiting spread of this species. The *M*. *graminis* species has already included in the government plant pest register.

## Conclusion

*Meloidogyne graminis* was identified causing damage to golf course turfgrass in the State of São Paulo. This is the first report of this species in Brazil, being its differential diagnosis based on sequence analyses from the D2-D3 and mtDNA COII regions and on phylogenetic analyses.
